# Gut microbiota in parasite-transmitting gastropods

**DOI:** 10.1186/s40249-023-01159-z

**Published:** 2023-11-24

**Authors:** Peipei Li, Jinni Hong, Zhanhong Yuan, Yun Huang, Mingrou Wu, Tao Ding, Zhongdao Wu, Xi Sun, Datao Lin

**Affiliations:** 1 Department of Parasitology, Zhongshan School of Medicine, Sun Yat-Sen University, Guangzhou, China; 2 Key Laboratory of Tropical Disease Control, Ministry of Education, Sun Yat-Sen University, Guangzhou, China; 3 Chinese Atomic Energy Agency Center of Excellence on Nuclear Technology Applications for Insect Control, Provincial Engineering Technology Research Center for Diseases-Vectors Control, Sun Yat-Sen University, Guangzhou, China; 4 Department of Traditional Chinese Medicine, Guangdong Provincial People’s Hospital, Guangdong Academy of Medical Sciences, Southern Medical University, Guangzhou, China

**Keywords:** Snail, Intermediate host, Gut microbiome, Potential application, Snail-born parasite, Schistosome, Schistosomiasis, *Angiostrongyluscantonensis*, Vector

## Abstract

**Background:**

Gastropoda, the largest class within the phylum Mollusca, houses diverse gut microbiota, and some gastropods serve as intermediate hosts for parasites. Studies have revealed that gut bacteria in gastropods are associated with various biological aspects, such as growth, immunity and host–parasite interactions. Here, we summarize our current knowledge of gastropod gut microbiomes and highlight future research priorities and perspectives.

**Methods:**

A literature search was undertaken using PubMed, Web of Science and CNKI for the articles on the gut microbiota of gastropods until December 31, 2022. We retrieved a total of 166 articles and identified 73 eligible articles for inclusion in this review based on the inclusion and exclusion criteria.

**Results:**

Our analysis encompassed freshwater, seawater and land snails, with a specific focus on parasite-transmitting gastropods. We found that most studies on gastropod gut microbiota have primarily utilized 16S rRNA gene sequencing to analyze microbial composition, rather than employing metagenomic, metatranscriptomic, or metabolomic approaches. This comprehensive review provided an overview of the parasites carried by snail species in the context of gut microbiota studies. We presented the gut microbial trends, a comprehensive summary of the diversity and composition, influencing factors, and potential functions of gastropod gut microbiota. Additionally, we discussed the potential applications, research gaps and future perspectives of gut microbiomes in parasite-transmitting gastropods. Furthermore, several strategies for enhancing our comprehension of gut microbiomes in snails were also discussed.

**Conclusions:**

This review comprehensively summarizes the current knowledge on the composition, potential function, influencing factors, potential applications, limitations, and challenges of gut microbiomes in gastropods, with a specific emphasis on parasite-transmitting gastropods. These findings provide important insights for future studies aiming to understand the potential role of gastropod gut microbiota in controlling snail populations and snail-borne diseases.

**Graphical abstract:**

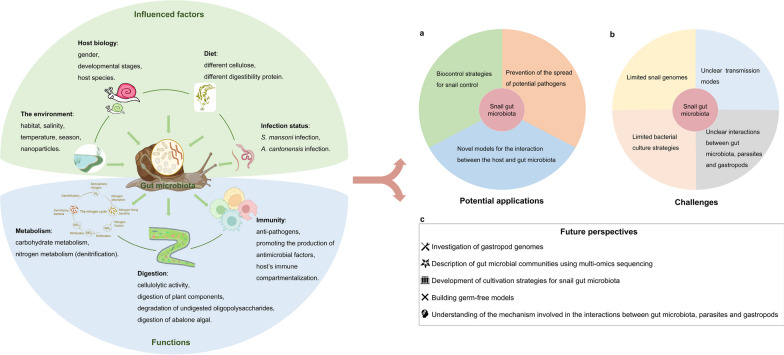

**Supplementary Information:**

The online version contains supplementary material available at 10.1186/s40249-023-01159-z.

## Background

Mollusca, the second largest phylum in the animal kingdom, represents one of the most diverse animal populations on Earth. Among the classes within this phylum, gastropods are the most widely distributed and abundant species, accounting for ~ 80% of all existing mollusk species [1]. Gastropods inhabit various environments including marine, freshwater, and terrestrial ecosystems, where they play vital roles in nutrient cycling, soil formation, productivity, and the decomposition of organic matter [2]. Importantly, certain gastropods serve as intermediate hosts for parasites such as *Schistosoma* *mansoni*, *S.* *japonica*, *S.* *haematobium*, *S.* *mekongi*, and *Angiostrongylus* *cantonensis*, contributing to the transmission of infectious diseases [3–7]. Additionally, parasite-transmitting snail *Biomphalaria* *straminea*, an important intermediate host of *S.* *mansoni*, has invaded Hong Kong, China, since 1974 and has spread in Guangdong, Southern China, which has garnered significant attention from various organizations, including disease control centers and universities [3, 8].

The emergence of invasive alien species (IAS) has become a pressing concern worldwide. These species refer to organisms unintentionally introduced and established beyond their native range, posing significant threats to human health, economy, biodiversity, and food security [9, 10]. Recently, certain gastropod species, such as *Pomacea* *canaliculata* and *Achatina* *fulica*, have gained particular attention due to their destructive impacts on ecosystems [11]. These invasive gastropods pose potential threats to public health, ecological environments, agriculture, and the economies of affected countries [6, 12, 13]. Given the substantial burdens caused by IAS, prioritizing monitoring and control efforts through effective strategies becomes crucial.

The gut microbiota refers to the trillions of microorganisms that inhabit the intestines [14]. Initially, our understanding of gut microbes in organisms was established through bacterial isolation and culture techniques. However, recent advancements in high-throughput sequencing have revolutionized our knowledge of gut microbiomes. Sequencing approaches such as metatranscriptome, metaproteome, and metabolome have provided deeper insights into gut microbiota composition [15]. Since the 1970s, researchers from various countries have been studying the gut microbiota of gastropods using bacterial isolation, 16S rRNA gene sequencing, and metagenomic sequencing.

Recent studies have shed light on the pivotal role of gut microbiota in host growth, development, adaptation to the environment, and interactions with pathogens [16–19]. Some research endeavors have explored the gut microbiomes of certain gastropod species [20–23]. These investigations have established correlations between gastropod gut microbiota and vital biological functions such as cellulose degradation and immune enhancement [13, 24]. Furthermore, scientists have endeavored to explore novel approaches such as modulating gut microbiota through diverse dietary interventions, aiming to develop potential control strategies for mitigating the spread of gastropods [23]. However, the biological characteristics underlying the interaction between gut microbiota and gastropods remain poorly understood. To address this issue, it is essential to explore the baseline of gut microbiota in gastropods and understand its influencing factors, potential functions, and the current limitations or challenges associated with gastropod gut microbiota. We believe that these efforts will contribute to further deepening our understanding of the potential application prospects of gastropod gut microbiota.

## Methods

### Information sources and search strategy

In this review, we searched the PubMed website (https://pubmed.ncbi.nlm.nih.gov/), Web of Science (https://www.webofscience.com/) and CNKI (China National Knowledge Infrastructure, https://www.cnki.net/) (In Chinese) for the articles on the gut microbiota of gastropods until December 31, 2022. For PubMed and Web of Science, the term used in the database search was ((snail) OR (gastropod)) AND ((gut microbiome) OR (gut microbiota) OR (intestinal microbiota)). For CNKI, the main subject term: (snail) AND (gut microbiota) was searched. We then searched for core journals in the CNKI Journal Evaluation System by selecting "Core Journal Library" or "Key Journal Library" from the drop-down menu on the CNKI homepage to identify core journals. For information on the search strategies used for the databases, please refer to Additional file 1. The included references were manually checked to determine the absence of additional studies.

### Eligibility criteria

Published papers were eligible for inclusion if they focused on the following inclusion criteria: (1) Gastropod gut microbiota; (2) Research articles; (3) Published before December 31, 2022; (4) Not duplicated studies; (5) “Core journal” screening at CNKI. Exclusion criteria for the present review consisted of: (1) Research on non-gastropod gut microbiota; (2) Review articles, opinion articles, letters, case reports, and conferences; (3) Duplicated studies; (4) Not a “core journal” at CNKI.

### Study screening, data extraction and analysis

This was then followed by a full-text review of identified articles, independently conducted by two reviewers. References identified in the initial studies were also reviewed and included in this review if they were deemed relevant. A comprehensive analysis was conducted for each eligible document, including a thorough review of the full-text and a narrative synthesis, which was then summarized into a qualitative review. Data extraction was completed by two researchers using a different pre-prepared checklist. The results of the screening and selection process are presented in Table 1.

**Table 1:** Comprehensive analysis of microbiomes in gastropods: insights from various snail species, habitat, source, publication year, and research techniques

Snail species	Habitat	Source	Publication year	Research techniques^a^	References
*Biomphalaria* *glabrata*	Freshwater	USA; USA; Brazil; USA; France; China; Brazil	1979; 1981; 2013; 2018; 2019; 2022; 2020	Isolation; isolation; isolation; 16S; 16S; metagenomic; 16S, respectively	[20, 23, 27–31]
*Cornu* *aspersum*	Freshwater	France; Bulgaria; USA; Greece; Greece	2006; 2014; 2019; 2020; 2019	Isolation; isolation; 16S; isolation; isolation, respectively	[24, 32–35]
*Helix* *pomatia*	Land	France	2006	Isolation	[24]
*Achatina* *fulica*	Land	Brazil; India; Brazil; China; Indonesia	2012; 2015; 2015; 2020; 2022	16S; Isolation; isolation; 16S, 16S, respectively	[13, 36–39]
*Achatinella* *mustelina*	Land	USA	2014	16S	[40]
*Potamopyrgus* *antipodarum*	Freshwater	New Zealand; UK	2016; 2021	16S; 16S, respectively	[41, 42]
*Turbo* *cornutus*	Seawater	Japan	2016	Isolation	[43]
*Rubyspira* *osteovora*	Seawater	USA	2017	16S	[44]
Cone snails	Seawater	USA	2017	16S	[45]
*Caracolus* *marginella*	Land	USA	2017	Metagenomic	[46]
*Rapana* *venosa*	Seawater	China; China; China; China	2018; 2019; 2020; 2022	16S; 16S; 16S and metabolomic, respectively	[47–50]
*Aplexa* *cf.* *marmorata*	Freshwater	Brazil	2018	16S	[21]
*Geomalacus* *maculosus*	Land	Ireland	2018	16S	[51]
*Radix* *auricularia*	Freshwater	China; China	2018; 2020	16S; 16S, respectively	[52, 53]
*Haliotis* *tuberculata*	Seawater	France	2018	16S	[54]
*Haliotis* *discus* *hannai*	Seawater	China; China; China	2018; 2018; 2022	16S; 16S, 16S, respectively	[55–57]
*Haliotis* *gigantea*	Seawater	Japan	2020	16S	[58]
*Haliotis* *fulgens*	Seawater	Mexico; Mexico	2018; 2018	16S; 16S, respectively	[59, 60]
*Haliotis* *corrugata*	Seawater	Mexico; Mexico	2018; 2018	16S; 16S, respectively	[59, 60]
*Batillus* *cornutus*	Seawater	Japan	2019	16S	[61]
*Oncomelania* *hupensis*	Freshwater	China	2020	16S	[62]
*Pomacea* *canaliculata*	Freshwater	China; China; China; China; China	2019; 2021; 2022; 2022; 2022	16S; 16S; 16S; 16S; 16S, respectively	[12, 63–66]
*Pomacea* *maculata*	Freshwater	China	2022	16S	[67]
*Theodoxus* *fluviatilis*	Freshwater	Germany	2020	16S	[68]
*Phyllocaulis* *soleiformis*	Land	Brazil	2020	16S	[31]
*Littorina* spp.	Seawater	Russian	2021	16S	[69]
*Planorbella* *trivolvis*	Freshwater	China; China	2021; 2020	16S; 16S, respectively	[22, 53]
*Oreohelix* *strigosa*	Land	USA; USA	2021; 2022	16S; 16S, respectively	[70, 71]
*Cipangopaludina* *chinensis*	Freshwater	China; China	2022; 2022	16S; 16S, respectively	[12, 72]
*Bradybaena* *ravida*	Land	China	2022	16S	[73]
*Bellamya* *aeruginosa*	Freshwater	China	2022	16S	[74]
*Ampullaceana* *balthica*	Freshwater	Estonia; Estonia	2022;2022	16S; 16S, respectively	[75, 76]
*Cipangopaludina* *cathayensis*	Freshwater	China	2022	16S	[77]
*Arion* *ater*	Land	UK; UK	2017; 2022	Metagenomic; metagenomic, respectively	[78, 79]
*Alviniconcha* *marisindica*	Seawater	China	2022	Metagenomic	[80]
*Trochus* *niloticus*	Seawater	China	2022	16S	[81]
*Indoplanorbis* *exustus*	Freshwater	India	2022	Isolation	[82]
*Juturnia* *kosteri*	Freshwater	USA	2022	16S	[83]
*Pyrgulopsis* *roswellensis*	Freshwater	USA	2022	16S	
*Ambigolimax* *valentianus*	Land	USA	2021	16S	[84]
*Alycaeus* *jagori*	land	Germany	2021	16S	[85]
*Georissa* *similis*					
*Plectostoma* *concinnum*					
*Diplommatina* *calvula*					
*Kaliella* *accepta*					
*Haliotis* *sorenseni*	Seawater	USA	2020	16S	[86]
*Lymnaea* *stagnalis*	Freshwater	UK	2020	16S	[87]
*Chlorostoma* *funebralis*	Seawater	USA	2019	16S	[88]
*Littorina* *keenae*					
*Lottia* *gigantea*					
*Benedictia* *baicalensis*	Freshwater	Russia	2018	16S	[89]
*Biomphalaria* *pfeifferi*	Freshwater	USA	2012	16S	[90]
*Bulinus* *africanus*					
*Helisoma* *duryi*					
*Cellana* *toreuma*	Seawater	Republic of Korea	2022	Isolation	[91]

^a^16S refers to 16S rRNA gene sequencing

### Quality assessment of included literature

We used the Joanna Briggs Institute Prevalence Critical Appraisal Tool to assess the quality of the included articles [25]. This tool consists of 10 quality control items, and each selected study was evaluated based on these criteria. For every item fulfilled, a score of one was assigned, while a zero score was given for unmet items. By aggregating the scores, we categorized the studies into three levels of quality: low (0–3), moderate (4–6), or high (7–10) [26]. For more detailed information, please refer to Additional file 2.

## Results

**Fig. 1:**
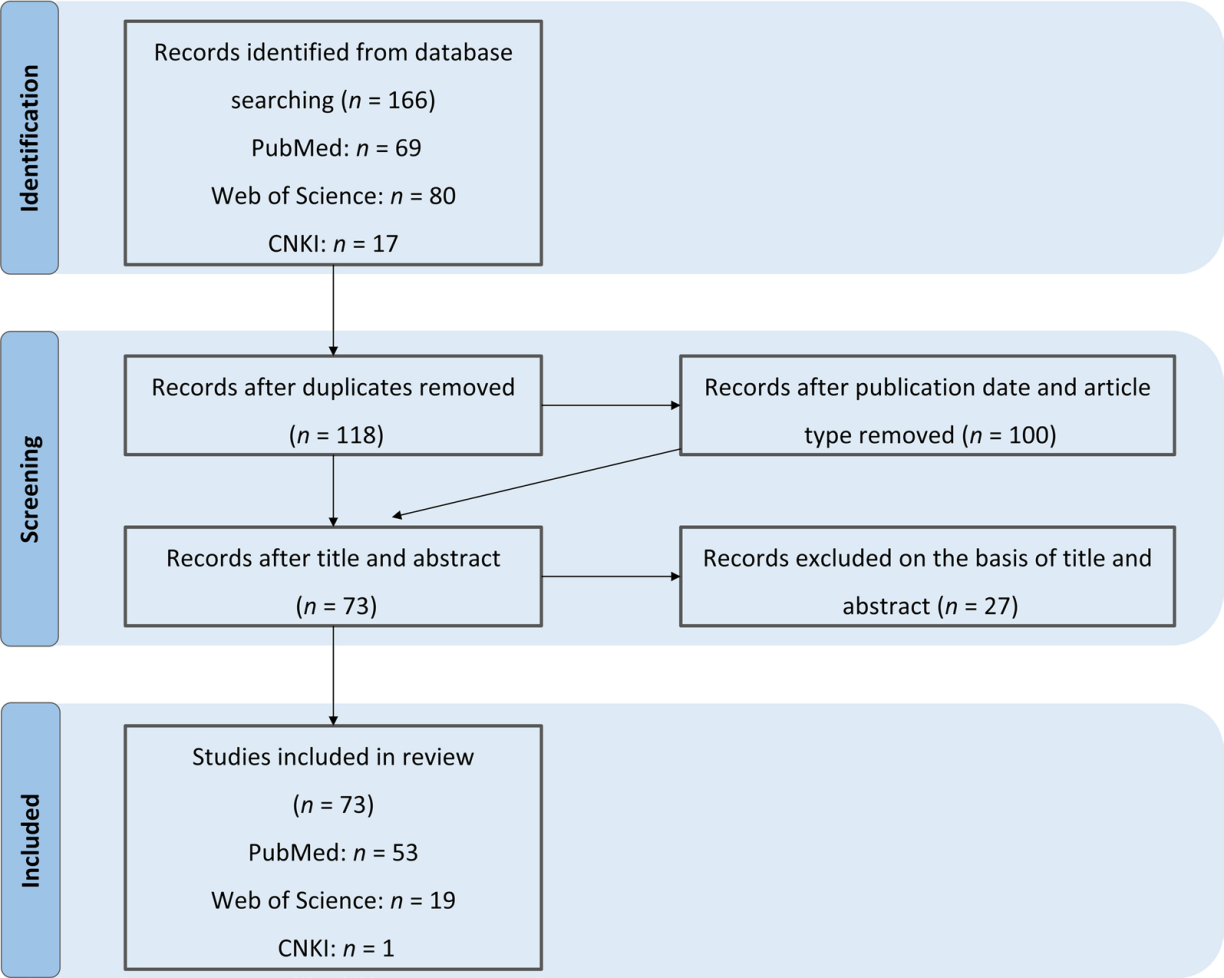
Flow diagram of literature screening and selection process

As shown in Fig. 1, we identified 166 relevant papers from electronic databases, comprising 69 from PubMed, 80 from Web of Science, and 17 from CNKI. After eliminating duplicates, considering publication dates and article types, 100 papers qualified for screening based on title and abstract relevance. At this stage, 27 papers were excluded, leaving 73 articles eligible for inclusion in this review.

## Trends in gut microbiota in gastropods

**Fig. 2:**
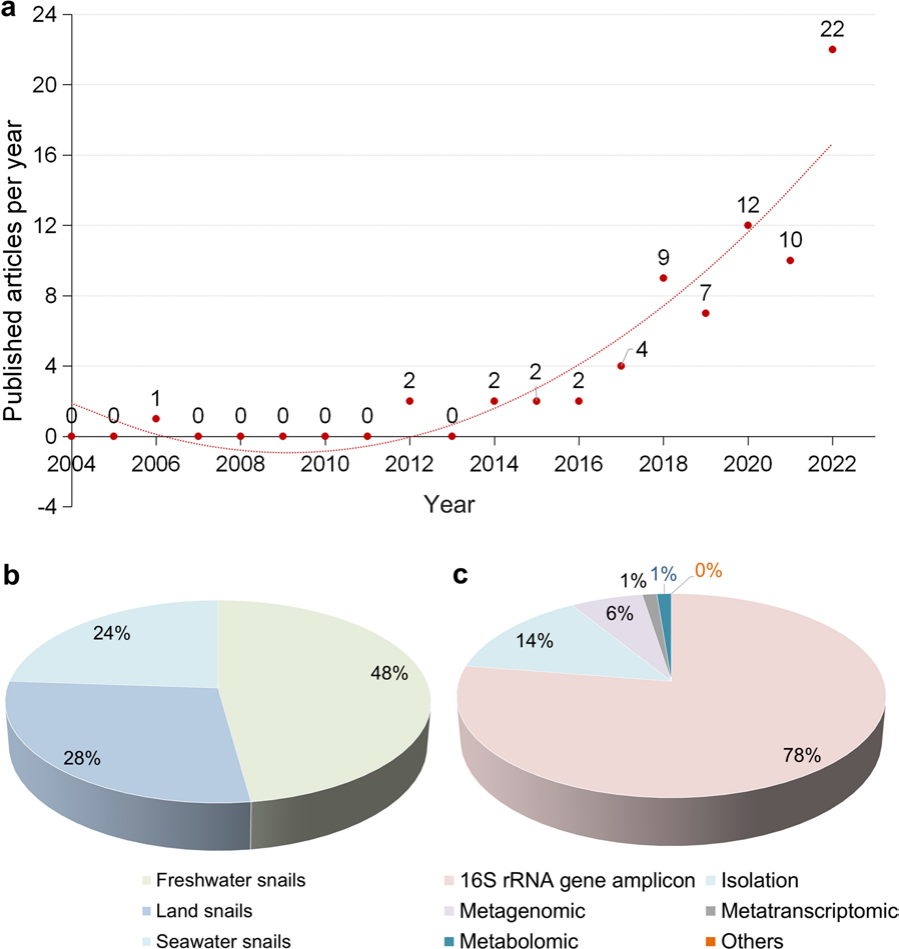
Published articles searched in PubMed (https://pubmed.ncbi.nlm.nih.gov/), Web of Science (https://www.webofscience.com/wos/woscc/basic-search), and CNKI (https://www.cnki.net/) databases by using the term words. **a** Number of research articles on the gut microbiota of gastropods between 2004 and 2022. **b** Distribution of publications by percentage, categorized according to land, freshwater, and seawater snail populations. **c** Percentage distribution of publications based on sequencing techniques used for studying the gastropod gut microbiota

Until 2012, there were limited studies on the gut microbiota of gastropods using high-throughput sequencing techniques in PubMed, Web of Science and CNKI databases (Fig. 2a). However, with advancements in technology and a better understanding of gastropod biology, more researchers have started focusing on the symbiotic bacteria present in the gut of gastropods. Overall, research on gastropod gut microbiota is rapidly growing.

Gastropods can be categorized into different populations based on their habitat, including land, freshwater, and seawater snails. The majority of publications on gastropod gut microbiota are related to freshwater snails, accounting for 48% (Fig. 2b). Furthermore, as depicted in Fig. 2c and Table 1, the most commonly used technique for studying gastropod gut microbiota is 16S rRNA gene sequencing, which constitutes 78% of the studies. This indicates that most research has focused on analyzing the microbial composition using 16S rRNA gene amplicon sequencing, rather than employing metagenomic, metatranscriptomic, or metabolomic approaches. However, it is worth considering combining multiple sequencing techniques in future studies to overcome the limitations of each individual method.

In addition, we specifically focused on gastropods that transmit parasites and act as intermediate hosts for various parasites. Many freshwater, land, and seawater snails, such as *Achatina* *fulica*, *Pomacea* *canaliculata* and *Haliotis* *tuberculata*, are responsible for transmitting parasites like *A.* *cantonensis*, *S.* *japonica*, *S.* *mansoni* and *Echinostoma* *cinetorchis* (Table 2). Some of these parasites are zoonotic and can cause diseases in humans. Therefore, this review specifically emphasized the biological aspects of the parasite-transmitting gastropod gut microbiota.

**Table 2:** Snails associated with gut bacterial studies and the snail-borne parasites

Snails	Parasites	Diseases	References
*Achatina* *fulica*	*Angiostrongylus* *cantonensis*	Angiostrongyliasis cantonensis	[92]
	*Angiostrongylus* *costaricensis*	Angiostrongyliasis costaricensis	[93]
	*Angiostrongylus* *malaysiensis*	–	[94]
	*Angiostrongylus* *vasorum*	Canine angiostrongylosis	[95]
	*Rhabditella* *axei*	Rhabditelliasis axei	[96]
	*Rhabditis* *terricola*	–	[96]
	*Pristionchus* *entomophagus*	–	[96]
	*Cruznema* spp.	–	[96]
*Bellamya* *aeruginosa*	*Angiostrongylus* *cantonensis*	Angiostrongyliasis cantonensis	[97]
	*Echinochasmus* *perfoliatus*	–	[98]
*Pomacea* *canaliculata*	*Angiostrongylus* *cantonensis*	Angiostrongyliasis cantonensis	[99]
	*Gnathostoma* *spinigerum*	Gnathostomiasis	[100]
	*Angiostrongylus* *vasorum*	Canine angiostrongylosis	[101]
*Pomacea* *maculata*	*Angiostrongylus* *cantonensis*	Angiostrongyliasis cantonensis	[102]
	*Stomylotrema* *gratiosus*	–	[103]
*Biomphalaria* *glabrata*	*Schistosoma* *mansoni*	Schistosomiasis	[104]
	*Angiostrongylus* *cantonensis*	Angiostrongyliasis cantonensis	[31]
	*Echinostoma* *caproni*	Echinostomiasis	[105]
*Cipangopaludina* *chinensis*	*Echinostoma* *cinetorchis*	Echinostomiasis	[106]
	*Angiostrongylus* *cantonensis*	Angiostrongyliasis cantonensis	[107]
*Cornu* *aspersum*	*Brachylaima* spp.	Brachylaimiasis	[108]
	*Angiostrongylus* *cantonensis*	Angiostrongyliasis cantonensis	[109]
*Oncomelania* *hupensis*	*Schistosoma* *japonicum*	Schistosomiasis japonica	[110]
	*Exorchis* spp.	–	[111]
*Phyllocaulis* *soleiformis*	*Angiostrongylus* *costaricensis*	Abdominal angiostrongyliasis	[112]
	*Angiostrongylus* *cantonensis*	Angiostrongyliasis cantonensis	[31]
*Radix* *auricularia*	*Fasciola*	Fasciolosis	[113]
	*Trichobilharzia* *franki*	Cercarial dermatitis	[114]
	Diplostomidae, Echinostomatidae, Notocotylidae, Plagiorchiidae, and Strigeidae	–	[115]
	*Diplostomum* *spathaceum,* *Paryphostomum* *radiatum,* *Echinoparyphium* *recurvatum,* *Opisthioglyphe* *ranae,* *Plagiorchis* *elegans,* *Australapatemon* *burti,* *Echinostomaspp.,* *Hypoderaeum* *conoideum,* *Isthmiophora* *melis,* *Notocotylus* *attenuatus,* *Tylodelphys* *clavata,* *Echinostoma* *revolutum,* *Trichobilharzia* *szidati*	–	[116]
*Potamopyrgus* *antipodarum*	*Atriophallophorus* *winterbourni*	–	[117]
	*Notocotylus* spp.	–	[117]
	*Aspidogaster* *conchicola*	–	[118]
	*Echinoparyphium* *aconiatum*	–	[118]
*Haliotis* *tuberculata*	*Haplosporidium* *montforti* n. spp.	–	[119]
*Littorina* spp.	*Himasthla* *elongata*	–	[120]
	*Renicola* *roscovita*	–	[121]
*Planorbella* *trivolvis*	*Neoechinorhynchus* *emydis*	–	[122]
	*Drepanocephalus* *spathans* spp.	–	[123]
	*Echinostoma* *trivolvis*	–	[124]
*Oreohelix* *strigosa*	*Brachylaime* *microti*	–	[125]
*Arion* *ater*	*Angiostoma* *norvegicum* n. spp.	–	[126]
	*Angiostrongylus* *vasorum*	Canine angiostrongylosis	[127]
*Biomphalaria* *pfeifferi*	*Schistosoma* *mansoni*	Schistosomiasis	[128]
*Bulinus* *africanus*	*Schistosoma* *haematobium*	Schistosomiasis	[129]
*Lymnaea* *stagnalis*	*Trichobilharzia* *szidati*	–	[130]
*Indoplanorbis* *exustus*	*Amphistome* *cercaria*	–	[131]
	*Echinostome* *cercaria*	–	[132]

“–” means not applicable

Overall, research on gastropod gut microbiota is rapidly expanding, with a particular focus on freshwater snails and the role of microbiota in parasite transmission. The utilization of high-throughput sequencing techniques and the integration of multiple sequencing methods hold great promise for future studies in this field.

## Gut microbial composition and diversity in parasite-transmitting gastropods

A total of 73 gastropod species have been studied for their gut microbiota as of December 2022 (Table 1). Among these, 20 snail species have been identified as capable of transmitting parasites (Table 2). The dominant phyla found in the gut microbiota of land and freshwater gastropods were Proteobacteria, Firmicutes, and Bacteroidetes [22, 31, 36, 75]. On the other hand, seawater gastropods showed dominance of the phyla Tenericutes, Proteobacteria, and Fusobacteria in their gut microbiota.

### Land gastropods

The giant African snail (*A.* *fulica*), known for transmitting various pathogens such as *A.* *cantonensis*, is highly invasive and found in many countries worldwide. A previous study showed that the crop of *A.* *fulica* harbored a higher abundance of Proteobacteria, while the fecal samples were dominated by Bacteroidetes and Firmicutes [36]. The gut microbiota of the European-protected slug *Geomalacus* *maculosus* from Ireland housed the highest relative abundance of Proteobacteria (73.1%), followed by Bacteroidetes (7.5%) [51]. *Phyllocaulis* *soleiformis*, an important intermediate host of *Angiostrongylus* *costaricensis* from Brazil, exhibited Proteobacteria, Bacteroidetes, and Verrucomicrobia as the core gut phyla [31]. *Oncomelania* *hupensis*, an intermediate host of *S.* *japonicum*, housed a diverse gut microbiota dominated by Actinobacteria, Proteobacteria, Firmicutes*,* and Bacteroidetes [62]. *Arion* *ater*, which transmits *Angiostoma* *norvegicum* n. spp. and *A.* *vasorum*, showed a high abundance of Proteobacteria in its gut, with Gammaproteobacteria being the majority [79].

### Freshwater gastropods

The composition of gut microbiota among various freshwater gastropods at the phylum level was similar. In the microbiome analysis of *B.* *glabrata* based on 16S rRNA gene sequencing, the core gut microbes were identified as Proteobacteria, Bacteroidetes, Cyanobacteria, and Planctomycetes [133]. *Cipangopaludina* *chinensis*, a widely distributed snail in Asia with high nutritional value and medicinal value, housed a high abundance of Proteobacteria and Verrucomibia in the guts, with the genus *Aeromonas* being the dominant bacterium [72]. *Planorbella* *trivolvis* from China showed Bacteroidetes and Proteobacteria as the most abundant phyla in its gut microbiota based on the 16S rRNA gene sequencing [22]. The gut microbiota of *Ampullaceana* *balthica* from Eurasia was dominated by Proteobacteria, Bacteroidetes, Planctomycetes, Actinobacteria, and Firmicutes [75].

### Seawater gastropods

The genus *Mycoplasma* was a common gut microbe among seawater gastropods. *Haliotis* *tuberculata*, which transmits *Haplosporidium* *montforti* n. spp., had *Psychrilyobacter*, *Mycoplasma*, and *Vibrio* as dominant bacteria in its gut [54]*.* *Littorina* spp., the intermediate hosts of *Himasthla* *elongata* and *Renicola* *roscovita*, harbored Proteobacteria and Fusobacteria in their gut [69]. The deep-sea snail *Rubyspira* *osteovora* in Monterey Canyon was dominated by gut microbes *Mycoplasma* and *Psychromonas* [44]. The gut microbiota analysis of *Haliotis* *discus* *hannai* from the Republic of Korea revealed dominant microbes Tenericutes and Fusobacteria in the guts. At the genus level, *Mycoplasma* was found to be the most abundant in the gut microbiota of *Haliotis* *discus* *hannai* [55]. Similarly, *Mycoplasma* was also the most abundant in the gut microbiota of *Rapana* *venosa* from China [47].

The gut microbiota composition of gastropods varies depending on the habitat and species, with certain phyla and genera being commonly found across different land, seawater and freshwater gastropods. Further investigations are urgently needed to understand the potential functional roles and ecological significance of these gut microorganisms in gastropod biological aspects and disease transmission.

## Potential functions of the gut microbiota in parasite-transmitting gastropods

The gut microbiota in parasite-transmitting gastropods serves important functions in host digestion, nutrient absorption, and overall health. Research has shown that certain microbial species, such as *Paraprevotella*, show the ability to recruit trypsin to their surface, leading to enhanced trypsin autolysis [134], and this process helps maintain gut homeostasis and can also impact the host's sensitivity to enteroviruses. Moreover, reducing the population of *Asaia* through rifampin treatment has been observed to delay the development of *Anopheles* *stephensi* larvae [16]. Here, this review highlights the role of gut microbes in host growth, development and resistance to pathogens of parasite-carrying gastropods.

### Metabolism and host digestion

The gut microbes in gastropods possess the ability to break down food components, aiding in the digestion process (Table 3). For instance, bacteria from the Actinobacteria group isolated from the digestive tract of *A.* *fulica* showed high cellulolytic activity and produced glycoside hydrolases [13]. Similarly, Proteobacteria strains isolated from the *A.* *fulica* intestine showed cellulase activity, contributing to the degradation of cellulose in the host’s diet [37].

**Table 3:** Dominant microbes and their potential functions in gastropods

Phylum	Genus	Snail species	Potential function	References
Proteobacteria	*Aeromonas* *Klebsiella* *Enterobacter*	*Achatina* *fulica*	Degrading cellulosic compounds	[13, 37]
	*Vibrio*	*Haliotis* *tuberculata*	Metabolizing cellulose and degrading extracellular oligosaccharides	[54]
	*Azonexus* *Acidovorax* *Rhodoferax* *Vogesella*	*Biomphalaria* *glabrata*	Carbohydrate metabolism; nitrogen metabolism	[23]
Actinobacteria	*Streptomyces*	*Achatina* *fulica*	Degrading cellulosic compounds	[13]
	*Cellulosimicrobium*			
	*Agromyces*			
	*Microbacterium*			
	*Nocardiopsis*			
Firmicutes	*Bacillus*	*Haliotis* *diversicolor*	Promoting immune status	[135]
	*Lactococcus*	*Pomacea* *canaliculata*	Antagonism against pathogens	[64]
	*Lactobacillus*	*Cornu* *aspersum*	Immunomodulation	[34]
Fusobacteria	*Psychrilyobacter*	*Haliotis* *discus* *hannai*	Degrading oligo-polysaccharide	[54]
Verrucomicrobia	–	*Biomphalaria* *glabrata*	Anti-inflammatory and immune-stimulant	[133]
Tenericutes	*Leuconostoc*	*Pomacea* *canaliculata*	Restoring intestinal disorder	[64]
Bacteroidetes	–	*Pomacea* *canaliculata*	Fermentative metabolism and degradation of oligosaccharides	[64]

“–” means not applicable

Additionally, through metagenomic sequencing, researchers have revealed that gut bacteria of *Cornu* *aspersum* and *Helix* *pomatia* are capable of degrading various plant components [24], suggesting that these microbial communities may play a critical role in the digestion of phytophagous snails. In the case of abalone, the dominant microbe in the digestive gland is the genus *Psychrilyobacter*, which is associated with the degradation of undigested oligopolysaccharides [135]. While abalones themselves may show limited ability to degrade complex polysaccharides, their gut microbes, such as *Vibrio*, are closely related to the digestion of abalone algal diet. These specific gut microbes can promote the breakdown of algae polysaccharides [54].

### Host immunity and protection against pathogens

**Fig. 3:**
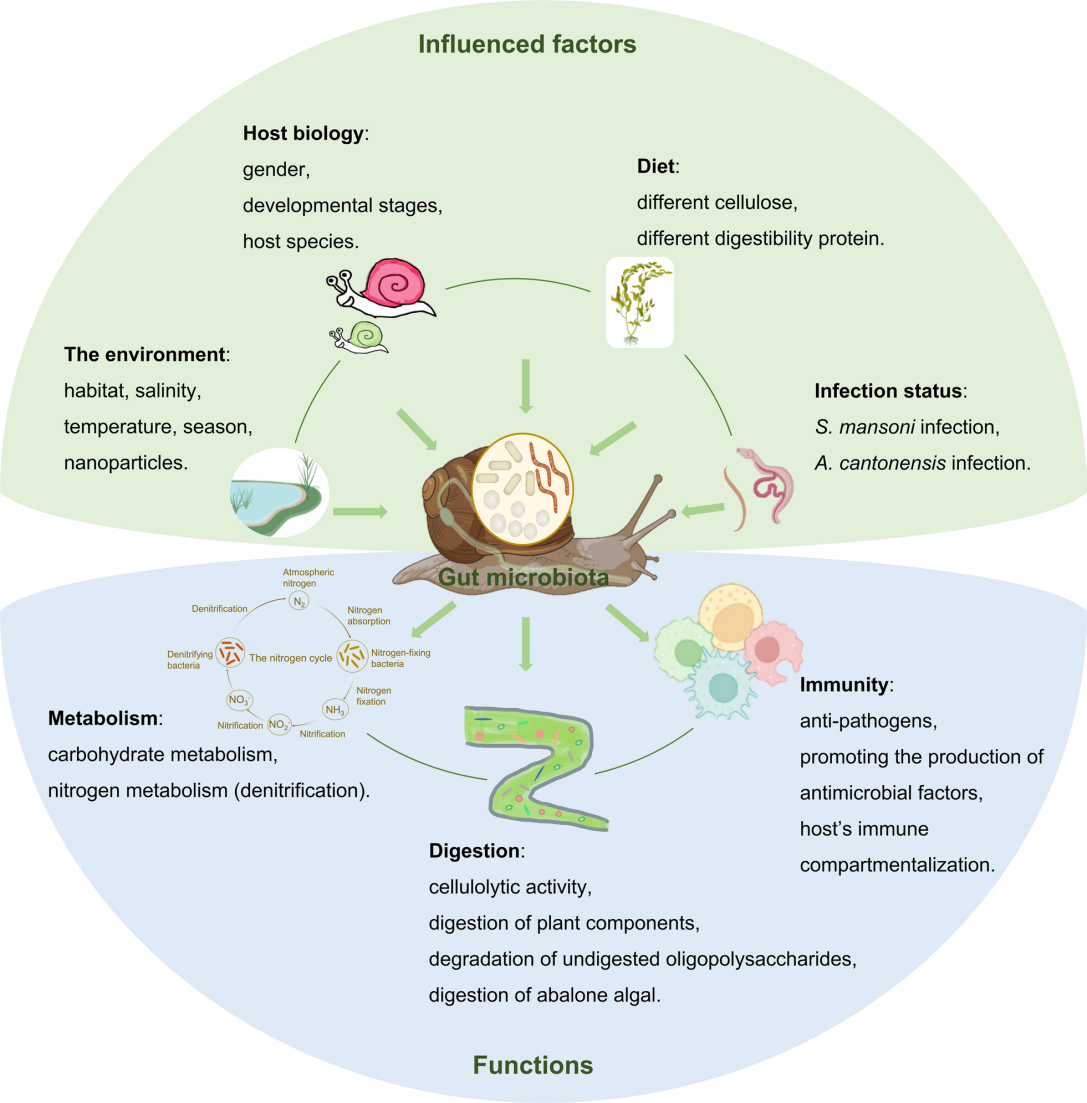
The potential functions and influencing factors of gastropod gut microbiota

The gut microbiota of parasite-transmitting gastropods plays a crucial role in host immunity (Table 3; Fig. 3). The host–microbiota homeostasis is associated with immune function and defense against bacterial pathogens [80]. Infection with parasites such as blood flukes (*S.* *mansoni*) led to changes in the composition and diversity of gut microbiota in mice [136]. Some bacterial species, such as Planctomycetes and Verrucomicrobia, showed significant alterations in abundance following infection, suggesting their potential involvement in interactions between the host and the parasite, as well as in maintaining the integrity of the intestinal barrier in parasite-transmitting gastropods [133]. Interestingly, Verrucomicrobia maintained higher abundance even 25 days post-infection, indicating its potential role in *S.* *mansoni*-infected *B.* *glabrata* [133]. Some strains of the *Lactococcus* isolated from *Arapaima* *gigas* fish could resist certain pathogenic bacteria, including *Citrobacter* *freundii*, *Pseudomonas* spp., *Enterobacter*, and *Aeromonas* *hydrophila*, suggesting that *Lactococcus* may be involved in the immunity of some pathogens in gastropods [64]. The Lactobacilli isolated from the *Cornu* *aspersum* gut tract demonstrated powerful inhibitory effects against *Salmonella* *enteritica* serotype Enteritis, *S.* *enteritica* serotype Choleraesuis and *Stapyloccocus* *epidermidis* [32]. Previous studies revealed that a strain of lactic acid bacteria isolated from *Cornu* *aspersum* can enhance the production of antimicrobial factors in the hemolymph and increase the bactericidal activity of snail serum against potential pathogens. Furthermore, dietary supplementation of the snail-gut commensal probiotic *Lactobacillus* *plantarum* Sgs14 strain has been found to reduce the mortality of *Listeria* *monocytogenes*-infected *Cornu* *aspersum* via exhibiting anti-Listeria activity [34].

In total, the gut microbiota of parasite-transmitting gastropods can play a critical role in host growth, development, and immunity to pathogens. These microbes are involved in host digestion and nutrient absorption, and can break down complex food components such as cellulose and plant components. Gut microbes may also play an important role in host immunity, maintaining gut homeostasis and defending against bacterial pathogens. The specific gut microbial communities present in parasite-carrying gastropods may hold significant potential for prospective applications. Further studies are urgently needed to fully elucidate the complex interactions between gut microbes and hosts, and to explore the potential role of these microorganisms in the transmission capacity of parasite-transmitting gastropods.

## Factors affecting the gut microbiota in parasite-transmitting gastropods

Both endogenous and exogenous factors can affect the gut microbiota of animals [137]. For instance, the parasite *Eimeria* can induce an imbalance in the gut microbes of chickens [138]. Significant differences in the composition of honeybee gut microbiota in different seasons were found [139]. The gut microbiota of seals is affected by age and sex [140]. In gastropods, the composition of gut microbiota can be affected by the environment, sex, diet, and infection status (Fig. 3), indicating that gastropod gut microbiota can be influenced by multiple factors.

### Habitats

The diversity and composition of microbial communities in gastropods' gut can vary based on their habitats. A study conducted by Li et al. [63] compared the microbiota in the buccal mass, stomach, and intestine of *P.* *canaliculata* and found that different sections of the digestive tract may show distinct dominant phyla, such as Bacteroidetes and Fusobacteria in the buccal mass, Cyanobacteria in the stomach, and Tenericutes and Spirochetes in the intestine. In *A.* *fulica*, Proteobacteria was the dominant phylum in the anterior segment of the digestive tract, while Bacteroidetes and Firmicutes were enriched in the fecal samples [36].

### Environmental factors

A comparative analysis of the gut microbiota of *O.* *hupensis* from four different ecological landscapes in Chinese mainland revealed that gastropods from marshlands and lakes showed the highest abundance of gut microbiota, while those from coastal areas displayed the lowest abundance. And the gut microbiota of gastropods from these landscapes showed significant differences at the genus level [62]. Bankers et al. [42] investigated the variation in the gut microbiota of *Potamopyrgus* *antipodarum* was compared between native populations in New Zealand and invaded populations in Europe. They found that the invasive gastropods housed more core microbes and higher species richness while the native retained a portion of their core microbiota. These results suggested that the living environment showed a great influence on the gut microbiota.

Salinity can also affect the composition of bacterial communities [141]. *Theodoxus* *fluviatilis* is capable of living in both fresh water and brackish water with salinities up to 28. Kivistik et al. [68] found significant differences in the composition of bacterial communities of *T.* *fluviatilis* under different salinity conditions.

Temperature and season are also key factors that affect the physiological state of animals [66, 142]. A study explored the effect of temperature on the gut microorganisms of *Rapana* *venosa*. *Mycoplasma* was the dominant genus, but the relative abundance among the three experimental groups differed noticeably. *Psychromonas* and *Vibrio* were only present in the low-temperature (16 ℃) group and the high-temperature (28 ℃) group, respectively, while *Flavobacteriaceae* was more abundant in the 22 ℃ and 28 ℃ groups [48]. The diversity of gut microbiota in *P.* *canaliculata* increased under high- and low-temperature conditions, although the composition of the core microbiome remained relatively unaffected [65]. Additionally, the gut microbial structure of *P.* *canaliculata* was significantly different among seasons [66].

Other factors, such as copper nanoparticles, could also affect the gut microbiota and protein profiles of *Indoplanorbis* *exustus* [82], indicating that copper nanoparticles may have implications for the health of snails and their gut microbiota. Additionally, exposure to cadmium had a significant impact on the community structure and function of gut microbiota, which could potentially affect the gut homeostasis and overall health of *Cipangopaludina* *cathayensis* [143].

### Host biology

A previous study suggested that both sex and developmental stages could affect the gut microbiota of *P.* *canaliculata*. The richness and diversity of gut microbiota were the highest in the female group and the lowest in the male group. In terms of community composition, the dominant gut microbial phyla of the female group are Proteobacteria, Actinobacteria and Chloroflexi, while Bacteroidetes and Tenericutes are abundant in the male group and juvenile group, respectively [64]. The composition of the gut microbiota was similar between young and adult *Cipangopaludina* *chinensis*, but the abundance of *Flavobacterium*, *Silanimonas*, *Geobacter* and *Zavarzinella* in young gastropods was significantly higher than that in adults [72]. Additionally, different snail species, such as *B.* *pfeifferi*, *Bulinus* *africanus* and *Helisoma* *duryi,* harbor distinct gut microbiota [90], indicating that host species play a role in shaping the gut microbiota of snails. In summary, gender, development stages and host species contribute to the variation in gastropod gut microbiota.

### Diet

Diets with varying levels of cellulose have been found to impact the gut microbiota and its metabolites. A study compared the gut microbiota of *Planorbella* *trivolvis* under different dietary conditions. It was observed that the relative abundance of Proteobacteria was 52.97% and Bacteroidetes was 28.75% in the cellulose-rich food group, while the relative abundance of Proteobacteria was 95.23% in the fiber-poor diet group, indicating that the fiber-poor diet significantly reduced the diversity of the gut microbiota in gastropods [22] Another study by Du et al. [23] used metagenomics sequencing to compare the differences in the gut microbiota of *B.* *glabrata* fed a low-digestibility protein and low polysaccharide diet (LPLP) versus a high-digestibility protein and high polysaccharide diet (HPHP). The results showed that *Chryseobacterium* was enriched in gastropods on the HPHP diet, while *Acidovorax* was enriched in gastropods on the LPLP diet. Furthermore, functional annotations showed that the HPHP group exhibited a higher abundance of carbohydrate-degrading genes, whereas the LPLP group had more denitrifying genes.

### Health status

The health status of gastropods affects their gut microbiota composition. The diversity of gut microbiota was found to be lower in diseased abalone compared to healthy abalone [144]. An analysis conducted by Portet et al. [133] investigated the changes in the microbiota of *B.* *glabrata* before and after *S.* *mansoni* infection and revealed that both the type and frequency of infection affect the snail microbiota. Of the core microbiota families, 69.4% were significantly affected by the infection. Tenericutes showed an increase after infection but decreased significantly after 4 days. Planctomycetes increased during primary infection but decreased significantly 1–4 days after reinfection. In the case of *A.* *cantonensis* infection, Proteobacteria in *B.* *glabrata* gut microbiota was increased while Nitrospirae and Tenericutes were decreased [31].

Combining this section, the gut microbiota of gastropods can be influenced by various endogenous and exogenous factors, such as habitats, environmental factors, host biology, diet and health status. The composition and diversity of microbial communities in the gut can vary based on the ecological landscapes in which gastropods reside, including salinity, temperature, and season. Host biology, particularly gender and developmental stage, as well as species-specific differences, can also shape the gut microbiota. Furthermore, dietary components, such as cellulose and protein, can impact the gut microbiota and its metabolites, while disease and infection can alter the gut microbiota composition as well. Understanding these factors and their impacts on gastropod gut microbiota will provide insight into the complex interactions between gastropods and their gut microbiomes.

## The application prospects of the gut microbiota in parasite-transmitting gastropods

**Fig. 4:**
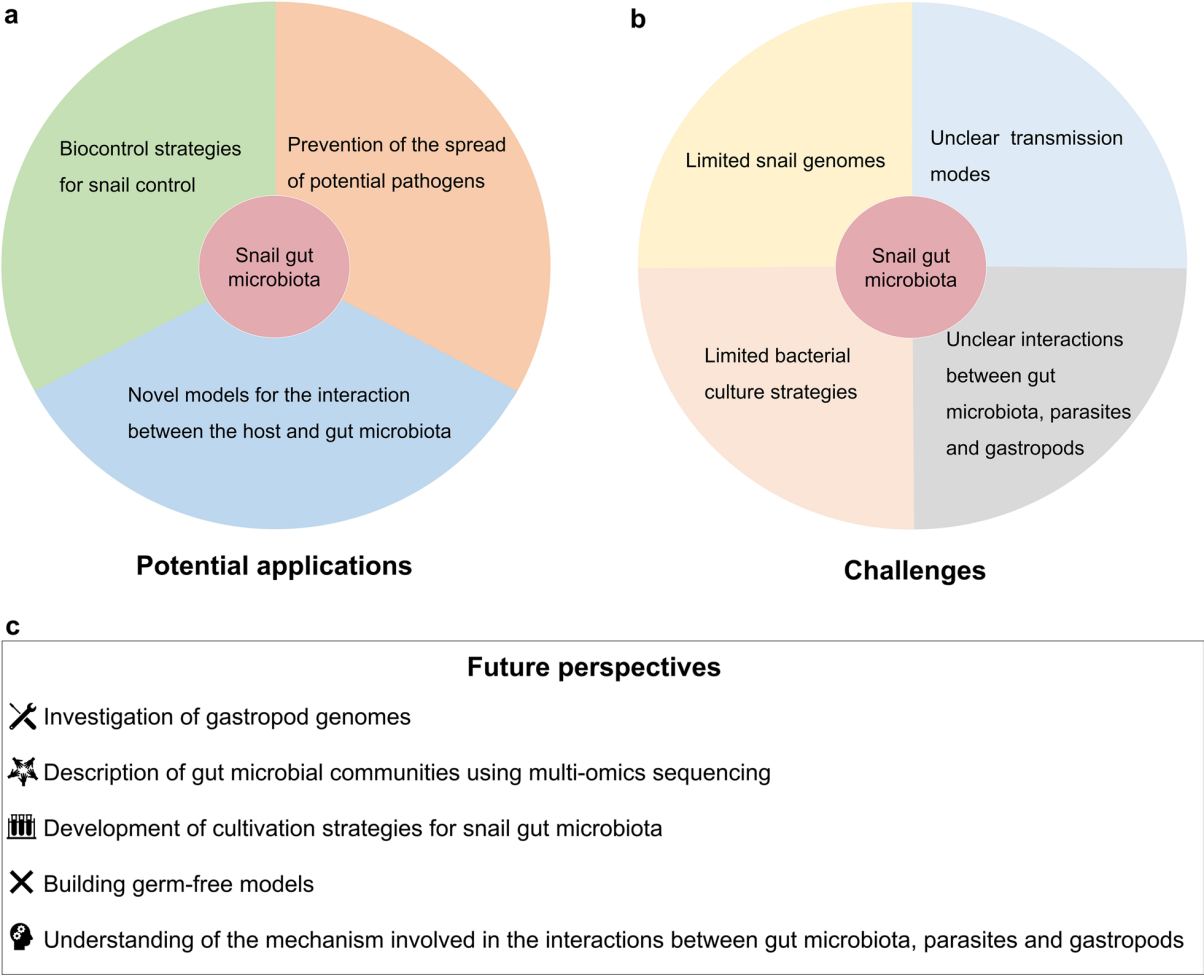
The potential applications (**a**), challenges (**b**) and future perspectives (**c**) of snail gut microbiota

In recent years, the potential applications of gut microbiota have been extensively explored in various fields, such as promoting human health [145], controlling insect populations [146], and modifying susceptibility to pathogens [147]. Researchers have also focused on the potential applications of gut microbiota in parasite-transmitting gastropods (Fig. 4a). The potential applications of gut microbiota in parasite-transmitting gastropods include controlling invasive gastropods, preventing the spread of parasites and studying host-microbiota interactions. Further studies are urgently needed to expand our knowledge of snail gut microbiota and its functional roles, as well as its potential applications in managing invasive gastropods and parasitic infections.

### Controlling the spread of invasive and parasite-transmitting gastropods

Invasive gastropods can cause serious impacts on human health, environmental ecology, and agricultural production. These gastropods can rapidly grow and reproduce, even under unfavorable conditions, leading to a reduction in the diversity of native species. Furthermore, some gastropods, such as *P.* *canaliculata* and *A.* *fulica*, serve as vectors for parasitic pathogens, posing a significant threat to human health [148–150]. To control the populations of invasive gastropods, traditional methods such as physical removal (such as manual collection and trapping) and chemical treatments (e.g., molluscicides like niclosamide) have been commonly used [151, 152]. However, these methods often pose challenges due to their potential inefficiency, high costs, and negative environmental impacts [5, 153]. Therefore, exploring biological control methods may present a promising alternative for managing invasive gastropods [5, 154, 155].

Previous studies have shown that disturbing the key gut microorganisms in insects can affect their survival, growth, and reproduction. For instance, female mosquitoes treated with antibiotics show decreased fecundity due to impaired digestion of blood proteins in the gut [156]. The *Wolbachia* *popcorn* strain has been found to shorten the lifespan of adult *Drosophila* *melanogaster* [157]. Similarly, disturbing the gut microbiota in honeybees can affect their gut metabolism, immunity, and overall survival rate [158]. Therefore, by studying the gut microbiota that plays a crucial role in snail physiology, we may gain insights into potential avenues for manipulating their microbiota to influence their survival, growth, and reproduction. For example, understanding the composition and function of the snail gut microbiota could help identify specific bacteria or microbial metabolites that are essential for snail health and development. This knowledge could be utilized to develop targeted probiotics or microbial-based biocontrol agents that promote beneficial microbial communities in snails, hindering the establishment of harmful pathogens or parasites. It's important to note that research on snail gut microbiota and its potential applications is still limited compared to insects like mosquitoes and honeybees. Further studies are needed to explore the diversity and functional roles of snail gut microorganisms, as well as their interactions with the snail host.

### Prevention of the spread of potential pathogens

Certain invasive gastropods, such as *A.* *fulica* [36] and *P.* *canaliculata* [150], serve as intermediate hosts for parasites such as *A.* *cantonensis*. Human infection can occur when consuming raw or undercooked gastropods. In insects, gut microorganisms such as *Serratia* *ureilytica* Su-YN1 found in mosquito midguts have been discovered to secrete enzymes that target and eliminate *Plasmodium* parasites [159]. This review highlights the significant threat that gastropods pose to human health as intermediate hosts for various parasites. Despite previous studies suggesting a relationship between changes in gut microbiota and parasite infection [31], it is crucial to conduct additional investigations to elucidate the specific mechanisms by which the gut microbiota of gastropods may influence parasite transmission. In summary, exploring the influence of gut microbiota in parasite-transmitting gastropods on their ability to transmit parasitic pathogens may offer valuable insights for advancing human health research.

### Snails as a novel model organism for studying host–microbiota interactions

Some snail species, such as *B.* *glabrata* and *B.* *straminea*, are easily cultured and manipulated in laboratory environments [5]. Their hermaphrodite characteristics may reduce interference from genetic factors during experiments [5]. Moreover, these snails can be infected with *S.* *mansoni*, making them an ideal model for investigating the interaction between microorganisms and parasitic infections [133]. Such research can enhance our understanding of how different microorganisms affect the susceptibility of hosts to parasites [31, 133, 160]. Therefore, these easily cultured and manipulated snail species, such as *B.* *glabrata* and *B.* *straminea*, with their hermaphrodite characteristics, provide an ideal model for studying the interaction between microorganisms and parasitic infections, particularly *S.* *mansoni*. By investigating this interaction, we can gain a better understanding of how different microorganisms influence the susceptibility of hosts to parasites.

Additionally, establishing a germ-free model of gastropods is an important endeavor that can greatly contribute to our understanding of the interactions between gut microbiota and hosts, similar to what has been done with mammals [161]. While certain techniques have been established for developing germ-free gastropods in a few species [20, 27], there are still limited strategies for establishing germ-free gastropods across various species. Only after successfully establishing a germ-free gastropod model can we better study the composition, function and interactions between the gastropod gut microbiota and the host. Therefore, there is an urgent need for further research in this area to refine the methods for establishing germ-free gastropods and to advance the study of gastropod gut microbiota.

## Limitations and prospects of studies on the gut microbiota in parasite-transmitting gastropods

In this study, we reviewed the composition and diversity, functions, and influencing factors of gastropod gut microbiota, and analyzed the potential applications of gut microbiomes in gastropods. However, several limitations and prospects that need to be addressed (Fig. 4b, c).

### Lack of sufficient gastropod genomes and limited application of novel sequencing technologies in snail gut microbiota

The development of sequencing technology has greatly contributed to our understanding of animal gut microbiota. For instance, metagenomic sequencing provided a more comprehensive composition of the gut microbiota compared to 16S rRNA gene sequencing. However, due to the limited availability of gastropod genomes, it is challenging to analyze the gut microbiota of gastropods without publicly available genomes using metagenomic, metatranscriptomic, or microbial single-cell RNA sequencing approaches.

### Dynamics and roles of gastropod gut microbiota: a limited understanding of temporal, spatial and generational changes

The transmission routes of gut microbiota play a crucial role in understanding the interactions between hosts and bacterial ecology. While studies on gut microbiota have largely focused on vertebrates and the transmission modes of commensal gut microbiota in mammals have been extensively studied [162], the routes of transmission of gastropod gut microbiota between hosts remain poorly understood. The baseline composition of the gut microbiota in major parasite-transmitting gastropods across China, such as *B.* *straminea*, *A.* *fulica* and *O.* *hupensis*, remains unclear. While we have discussed various factors such as sex, diet, habitat, infection status, and host genetics that can affect the gut microbiota of snails, the major factors responsible for shaping the gut microbiomes of snails remain unclear. Additionally, while the gut microbiota can influence fitness in mammals, the associations between gut microbiota and gastropod fitness, as well as the impact of gut microbiota on gastropod adaptation to the environment, are still poorly understood.

### Inadequate investigations of gut microbiota–parasites–gastropod interactions

The gut microbiota of animals is influenced by various external factors, such as diet, environment and parasitic infection. Although previous studies have provided a general understanding of gut bacteria in gastropods, they were insufficient to comprehensively understand the gut microbial composition of snails and to explore the relationship between snail gut microbiota and environmental factors. Moreover, numerous gastropod species can be infected by multiple parasites such as *S.* *mansoni*, and there is a lack of accurate methods to analyze the infection status of gastropods. Therefore, further studies are needed to determine whether the gut microbiota can serve as a potential alternative tool for distinguishing the infection status of wild gastropods.

### Limited development of bacterial culture techniques in gastropods

Bacterial culture techniques have been instrumental in obtaining mammalian gut microbes, such as *Bacteroides* *fragilis*, which were proven to participate in host immunity [163]. Furthermore, a previous study significantly improved researchers' ability to isolate pure cultures and identify new taxa, thus aiding in the conservation and utilization of microbial resources from various extreme environments [164]. Earlier investigations have explored the influence of various diets on the gut microbiota of *Helix* *aspersa* snails, revealing a prevalent Gram-positive coccobacillus (*Enterococcus* *casseliflavus*) under anaerobic conditions, and indicating potential synergistic interactions between the snail and bacteria [165]. However, the development of bacterial cultivation strategies to further reveal the real need for nutrients and the suitability of environmental factors that ensure the growth of the microorganisms to be isolated in snails is still limited. Additional studies are urgently necessary to determine which gastropod gut microorganisms can be cultured successfully and which play key roles in important biological aspects, such as development and immunity.

Taking together, several challenges and limitations need to be addressed in the study of gastropod gut microbiota. These include the lack of available gastropod genomes for metagenomic sequencing, inadequate understanding of the temporal and spatial changes in gut microbiota, limited investigations of gut microbiota-parasites-gastropod interactions, and the limited development of bacterial culture techniques in gastropods. We believe that addressing these challenges will lead to a more comprehensive understanding of gastropod gut microbiota and its potential applications in controlling invasive gastropods and preventing the spread of parasites.

## Conclusions

Gastropods, which play a crucial role in ecosystems and serve as intermediate hosts for numerous parasites, have a limited understanding of their gut microbiota. There is an urgent need for further investigation into the gastropod gut microbiota, as it plays an important role in the biological aspects of gastropods and the transmission of potential parasitic pathogens. This article is divided into five sections: the trends in gut microbiota of gastropods, gut microbial composition and diversity, potential functions of gut microbiota, the factors affecting the gut microbiota, and the application prospects of the gut microbiota. The limitations and challenges in this field were also discussed. These sections provide a comprehensive overview of the current knowledge and form the foundation for future research aimed at understanding the biological characteristics underlying the interaction between the gut microbiota and gastropods, as well as preventing the spread of parasites. Exploring novel strategies to modulate gut microbiota for controlling invasive gastropods may hold potential applications in mitigating snail-borne diseases and safeguarding human health.

### Supplementary Information

**Additional file 1**: Information sources and search strategy.

**Additional file 2**: Quality assessment of included literature.

## Data Availability

The datasets used and/or analyzed during the current study are available from the corresponding author on reasonable request.

## Abbreviations

*IAS*: Invasive alien species
*LPLP*: Low-digestibility protein and low polysaccharide diet
*HPHP*: Ligh-digestibility protein and high polysaccharide diet
