# Supplementary material for: Gut microbiota in parasite-transmitting gastropods
Source: Infect Dis Poverty. 2023 Nov 24;12:105. doi: 10.1186/s40249-023-01159-z (PMC10668521; doi:10.1186/s40249-023-01159-z)
Supplement: Supplementary file 1 — Additional file 1: Information sources and search strategy. [file 40249_2023_1159_MOESM1_ESM.docx]

**Additional file 1:** Information sources and search strategy

| **a. PubMed Search Query**  ((snail) OR (gastropod)) AND ((gut microbiome) OR (gut microbiota) OR (intestinal microbiota)) |
| --- |
| **b. Web of Science Search Query**  ((snail) OR (gastropod)) AND ((gut microbiome) OR (gut microbiota) OR (intestinal microbiota)) |
| **c. CNKI Search Strategy**  (螺) AND (肠道菌群) |

螺 = snail; 肠道菌群 = gut microbiota
