# Supplementary material for: Gut microbiota in parasite-transmitting gastropods
Source: Infect Dis Poverty. 2023 Nov 24;12:105. doi: 10.1186/s40249-023-01159-z (PMC10668521; doi:10.1186/s40249-023-01159-z)
Supplement: Supplementary file 2 — Additional file 2: Quality assessment of included literature. [file 40249_2023_1159_MOESM2_ESM.docx]

**Additional file 2:** Quality assessment of included literature

The quality of the included articles was assessed using the Joanna Briggs Institute (JBI) Prevalence Critical Appraisal Tool [1]. The tool assessed each article on the following:

1. Sample representative of the target population
2. Study participants recruited in an appropriate way
3. Sample size adequate
4. Study subjects and setting described in detail
5. Data analysis was conducted with enough coverage of the identified sample
6. Validity of methods used to identify the condition of interest
7. Condition measured in a standard, reliable way for all participants
8. Appropriate statistical analysis
9. Adequacy of response rate; were low response rates managed?
10. Sub-population of interest identified using objective criteria

| Author(s) and Year | Was the sample representative of the target population? | Were study participants recruited in an appropriate way? | Was the sample size adequate? | Were the study subjects and setting described in detail? | Was data analysis conducted with sufficient coverage of the identified sample? | Were valid methods used for the identification of the condition? | Was the condition measured in a standard, reliable way for all participants? | Was there appropriate statistical analysis? | Was the response rate adequate, and if not, was the low response rate managed appropriately? | Were target subpopulations identified using objective criteria? | Quality score |
| --- | --- | --- | --- | --- | --- | --- | --- | --- | --- | --- | --- |
| Zhou Z et al., 2022 | Yes | Yes | Yes | Yes | Not specified | Not specified | Not specified | Yes | NA | Not specified | 5 |
| Pinheiro GL et al., 2015 | Yes | Yes | No | Yes | Not specified | Not specified | Not specified | Yes | NA | Not specified | 4 |
| Ducklow HW et al., 1979 | Yes | Yes | Yes | Yes | Not specified | Not specified | Not specified | No | NA | Not specified | 3 |
| Lyra ML et al., 2018 | Yes | Yes | Yes | Yes | Not specified | Not specified | Not specified | Yes | NA | Not specified | 5 |
| Hu Z et al., 2021 | Yes | Yes | Yes | Yes | Not specified | Not specified | Not specified | Yes | NA | Not specified | 5 |
| Du S et al., 2022 | Yes | Yes | Yes | Yes | Not specified | Not specified | Not specified | Yes | NA | Not specified | 5 |
| Charrier M et al., 2006 | Yes | Yes | Yes | Yes | Not specified | Not specified | Not specified | Yes | NA | Not specified | 5 |
| Ducklow HW et al., 1981 | Yes | Yes | Yes | Yes | Not specified | Not specified | Not specified | No | NA | Not specified | 3 |
| Silva TM et al., 2013 | Yes | Yes | Yes | Yes | Not specified | Not specified | Not specified | No | NA | Not specified | 3 |
| Allan E et al., 2018 | Yes | Yes | Yes | Yes | Not specified | Not specified | Not specified | Yes | NA | Not specified | 5 |
| Huot C et al., 2019 | Yes | Yes | Yes | Yes | Not specified | Not specified | Not specified | Yes | NA | Not specified | 5 |
| Osorio JB et al., 2020 | Yes | Yes | Yes | Yes | Not specified | Not specified | Not specified | Yes | NA | Not specified | 5 |
| Koleva Z et al., 2014 | Yes | Yes | Yes | Yes | Not specified | Not specified | Not specified | No | NA | Not specified | 3 |
| Smith PN et al., 2019 | Yes | Yes | Yes | Yes | Not specified | Not specified | Not specified | Yes | NA | Not specified | 5 |
| Dushku E et al., 2020 | Yes | Yes | Yes | Yes | Not specified | Not specified | Not specified | No | NA | Not specified | 3 |
| Dushku E et al., 2019 | Yes | Yes | Yes | Yes | Not specified | Not specified | Not specified | No | NA | Not specified | 3 |
| Cardoso AM et al., 2012 | Yes | Yes | Yes | Yes | Not specified | Not specified | Not specified | Yes | NA | Not specified | 5 |
| Pawar KD et al., 2015 | Yes | Yes | Yes | Yes | Not specified | Not specified | Not specified | Yes | NA | Not specified | 5 |
| Song Y et al., 2020 | Yes | Yes | Yes | Yes | Not specified | Not specified | Not specified | Yes | NA | Not specified | 5 |
| Ni'Matuzahroh et al.,2022 | Yes | Yes | Yes | Yes | Not specified | Not specified | Not specified | Yes | NA | Not specified | 5 |
| O'Rorke R et al., 2015 | Yes | Yes | Yes | Yes | Not specified | Not specified | Not specified | Yes | NA | Not specified | 5 |
| Takacs-Vesbach C et al., 2016 | Yes | Yes | Yes | Yes | Not specified | Not specified | Not specified | Yes | NA | Not specified | 5 |
| Bankers L et al., 2021 | Yes | Yes | Yes | Yes | Not specified | Not specified | Not specified | Yes | NA | Not specified | 5 |
| Doi H et al., 2016 | Yes | Yes | Yes | Yes | Not specified | Not specified | Not specified | Yes | NA | Not specified | 5 |
| Aronson HS et al., 2017 | Yes | Yes | Yes | Yes | Not specified | Not specified | Not specified | Yes | NA | Not specified | 5 |
| Torres JP et al., 2017 | Yes | Yes | Yes | Yes | Not specified | Not specified | Not specified | Yes | NA | Not specified | 5 |
| Rabelo-Fernandez RJ et al., 2018 | Yes | Yes | Yes | Yes | Not specified | Not specified | Not specified | Yes | NA | Not specified | 5 |
| Song H et al., 2018 | Yes | Yes | Yes | Yes | Not specified | Not specified | Not specified | Yes | NA | Not specified | 5 |
| Yang MJ et al., 2019 | Yes | Yes | Yes | Yes | Not specified | Not specified | Not specified | Yes | NA | Not specified | 5 |
| Yang M et al., 2020 | Yes | Yes | Yes | Yes | Not specified | Not specified | Not specified | Yes | NA | Not specified | 5 |
| Yang M et al., 2022 | Yes | Yes | Yes | Yes | Not specified | Not specified | Not specified | Yes | NA | Not specified | 5 |
| Reich I et al., 2018 | Yes | Yes | Yes | Yes | Not specified | Not specified | Not specified | Yes | NA | Not specified | 5 |
| Hu Z et al., 2018 | Yes | Yes | Yes | Yes | Not specified | Not specified | Not specified | Yes | NA | Not specified | 5 |
| Hu Z et al., 2020 | Yes | Yes | Yes | Yes | Not specified | Not specified | Not specified | Yes | NA | Not specified | 5 |
| Gobet A et al., 2018 | Yes | Yes | Yes | Yes | Not specified | Not specified | Not specified | Yes | NA | Not specified | 5 |
| Nam BH et al., 2018 | Yes | Yes | Yes | Yes | Not specified | Not specified | Not specified | Yes | NA | Not specified | 5 |
| Huang Z et al., 2020 | Yes | Yes | Yes | Yes | Not specified | Not specified | Not specified | Yes | NA | Not specified | 5 |
| Yu X et al., 2022 | Yes | Yes | Yes | Yes | Not specified | Not specified | Not specified | Yes | NA | Not specified | 5 |
| Mizutani Y et al., 2020 | Yes | Yes | Yes | Yes | Not specified | Not specified | Not specified | Yes | NA | Not specified | 5 |
| Cicala F et al., 2018 | Yes | Yes | Yes | Yes | Not specified | Not specified | Not specified | Yes | NA | Not specified | 5 |
| Cicala F et al., 2018 | Yes | Yes | Yes | Yes | Not specified | Not specified | Not specified | Yes | NA | Not specified | 5 |
| Ito M et al., 2019 | Yes | Yes | Yes | Yes | Not specified | Not specified | Not specified | Yes | NA | Not specified | 5 |
| Hao Y et al., 2020 | Yes | Yes | Yes | Yes | Not specified | Not specified | Not specified | Yes | NA | Not specified | 5 |
| Li LH et al., 2019 | Yes | Yes | Yes | Yes | Not specified | Not specified | Not specified | Yes | NA | Not specified | 5 |
| Chen L et al., 2021 | Yes | Yes | Yes | Yes | Not specified | Not specified | Not specified | Yes | NA | Not specified | 5 |
| Li S et al., 2022 | Yes | Yes | Yes | Yes | Not specified | Not specified | Not specified | Yes | NA | Not specified | 5 |
| Li S et al., 2022 | Yes | Yes | Yes | Yes | Not specified | Not specified | Not specified | Yes | NA | Not specified | 5 |
| Li S et al., 2022 | Yes | Yes | Yes | Yes | Not specified | Not specified | Not specified | Yes | NA | Not specified | 5 |
| Kivistik C et al., 2020 | Yes | Yes | Yes | Yes | Not specified | Not specified | Not specified | Yes | NA | Not specified | 5 |
| Maltseva AL et al., 2021 | Yes | Yes | Yes | Yes | Not specified | Not specified | Not specified | Yes | NA | Not specified | 5 |
| Chalifour B et al., 2021 | Yes | Yes | Yes | Yes | Not specified | Not specified | Not specified | Yes | NA | Not specified | 5 |
| Chalifour BN et al., 2022 | Yes | Yes | Yes | Yes | Not specified | Not specified | Not specified | Yes | NA | Not specified | 5 |
| Zhou K et al., 2022 | Yes | Yes | Yes | Yes | Not specified | Not specified | Not specified | Yes | NA | Not specified | 5 |
| Zhang P et al., 2022 | Yes | Yes | Yes | Yes | Not specified | Not specified | Not specified | Yes | NA | Not specified | 5 |
| Lyu T et al., 2022 | Yes | Yes | Yes | Yes | Not specified | Not specified | Not specified | Yes | NA | Not specified | 5 |
| Kivistik C et al., 2022 | Yes | Yes | Yes | Yes | Not specified | Not specified | Not specified | Yes | NA | Not specified | 5 |
| Kivistik C,et al., 2022 | Yes | Yes | Yes | Yes | Not specified | Not specified | Not specified | Yes | NA | Not specified | 5 |
| Wu YY et al., 2022 | Yes | Yes | Yes | Yes | Not specified | Not specified | Not specified | Yes | NA | Not specified | 5 |
| Madubuike H et al., 2022 | Yes | Yes | Yes | Yes | Not specified | Not specified | Not specified | Yes | NA | Not specified | 5 |
| Joynson R et al., 2017 | Yes | Yes | Yes | Yes | Not specified | Not specified | Not specified | Yes | NA | Not specified | 5 |
| Yang Y et al., 2022 | Yes | Yes | Yes | Yes | Not specified | Not specified | Not specified | Yes | NA | Not specified | 5 |
| Zhang T et al., 2022 | Yes | Yes | Yes | Yes | Not specified | Not specified | Not specified | Yes | NA | Not specified | 5 |
| Mehraj A et al., 2022 | Yes | Yes | Yes | Yes | Not specified | Not specified | Not specified | Yes | NA | Not specified | 5 |
| Walters AD et al., 2022 | Yes | Yes | Yes | Yes | Not specified | Not specified | Not specified | Yes | NA | Not specified | 5 |
| Jackson D et al., 2021 | Yes | Yes | Yes | Yes | Not specified | Not specified | Not specified | Yes | NA | Not specified | 5 |
| Hendriks KP et al., 2021 | Yes | Yes | Yes | Yes | Not specified | Not specified | Not specified | Yes | NA | Not specified | 5 |
| Parker-Graham CA et al., 2020 | Yes | Yes | Yes | Yes | Not specified | Not specified | Not specified | Yes | NA | Not specified | 5 |
| Horton AA et al., 2020 | Yes | Yes | Yes | Yes | Not specified | Not specified | Not specified | Yes | NA | Not specified | 5 |
| Neu AT et al., 2019 | Yes | Yes | Yes | Yes | Not specified | Not specified | Not specified | Yes | NA | Not specified | 5 |
| Shtykova YR et al., 2018 | Yes | Yes | Yes | Yes | Not specified | Not specified | Not specified | Yes | NA | Not specified | 5 |
| Van Horn DJ et al., 2012 | Yes | Yes | Yes | Yes | Not specified | Not specified | Not specified | Yes | NA | Not specified | 5 |
| Yoo JH et al., 2022 | Yes | Yes | Yes | Yes | Not specified | Not specified | Not specified | No | NA | Not specified | 5 |

**Reference**

1. Munn Z, Moola S, Riitano D, Lisy K. The development of a critical appraisal tool for use in systematic reviews addressing questions of prevalence. Int J Health Policy Manag. 2014;3(3):123-8.
